# Preclinical and clinical issues in Alzheimer's disease drug research and development

**DOI:** 10.3389/fphar.2014.00234

**Published:** 2014-10-28

**Authors:** Cesare Mancuso, Silvana Gaetani

**Affiliations:** ^1^Institute of Pharmacology, Catholic University School of MedicineRome, Italy; ^2^Department of Physiology and Pharmacology “Vittorio Erspamer,” Sapienza University of RomeRome, Italy

**Keywords:** Alzheimer's disease, preclinical studies, clinical trials as topic, drug research and development, neurodegeneration

Alzheimer's disease (AD) is a chronic, rapidly progressive neurodegenerative disease, characterized by loss of memory and cognitive abilities, inability to carry out regular daily activities and mental disorders. The following numbers provide an idea of the extent of the problem: 36 million people worldwide suffer from dementia, of which 7.7 million new cases each year, and 60–70% of them are diagnosed with AD. Patients with AD deal with numerous problems, one of the most relevant being the lack of drugs that can slow down or hinder disease onset. Indeed, the currently available drugs, namely acetylcholinesterase inhibitors donepezil, rivastigmine, and galantamine, and the NMDA receptor antagonist, memantine, are able to counteract or delay the symptoms related to cognitive decline and nearly entirely in individuals with mild-to-moderate AD. The shortage of drugs available for the treatment of AD is also related to the difficulties faced by many pharmaceutical companies, including some Big Pharma, that despite significant achievements from their hit/leads in preclinical and early stages of clinical research, have not been able in recent years, to launch their products on the market due to ineffective results in terms of efficacy or safety of advanced clinical trials. This problem is at least in part due to investigators' difficulties in identifying the most adequate outcomes in order to plan clinical trials. Indeed, in many cases, attention has been focused on certain plasma or cerebral-spinal fluid (CSF) biomarkers, both for the relative ease of sampling and for the possibility they could offer to monitor the disease progression. However, it is worth emphasizing, that despite clinical trials based on the use of peripheral biomarkers have provided encouraging results on the ability of some drugs to block the production or deposition of β-amyloid (Aß) or even destroy existing senile plaques, key phase III studies, specifically designed to evaluate the improvement of cognitive activity, have shown a reduced ability of these new molecules to enhance memory or the ability to perform regular daily living functions in patients with AD or, in some cases have also resulted in severe side effects. Another limitation of pharmacological research on AD is the scarce ability to enhance collaboration between pharmaceutical industries and public or private non-profit organizations conducting basic research, such as universities. Both these sectors would benefit from the strengthening of this cooperation allowing non-profit institutions to enhance basic research on AD by receiving funds from pharmaceutical companies that, in turn, could benefit from the research of qualified scientists worldwide.

This Research Topic has been designed as a platform to analyze some of the most relevant problems posed by research on AD, suggest and develop new ideas that may improve both preclinical and clinical research on this disease. This Research Topic in which leading experts in the field of AD have contributed, is organized in 5 review articles, 3 original research articles, 1 opinion article, 1 Perspective article and 1 Commentary.

The complexity of the events that determine the onset and progression of AD is emphasized by the many factors that contribute to the pathogenesis of this disease. Recently, what has been pointed out is the pathogenetic role played by posttranslational modifications, such SUMOylation, on proteins involved in the production of Aß (Nisticò et al., 2014). In light of this, it is worth noting the remarkable discovery in Tg2576 transgenic mice, a validated preclinical model for the study of AD, that the imbalance of the phenomena of protein SUMOylation/deSUMOylation occurs in cognitive brain areas, such as the hippocampus and cortex, in the very early stages of the disease. This finding certainly contributes to indicate an alternative target for the development of new drugs for AD (Nisticò et al., 2014). The importance of having predictive preclinical models for the proper study of AD is also pointed out by Davis and Head (2014) who suggest the aged canine as an useful animal species for studies on AD and as an alternative to rodent models. Indeed, it has been shown that elderly beagle dogs have a progressive loss of cognitive functions with age and they show characteristic neuropathological hallmarks of AD (Davis and Head, 2014). This canine model allowed preclinical studies on the role of neuroprotective drugs, such as statins. An interesting link between the pathogenesis of AD and of other, apparently different, diseases, such as glaucoma, has been highlighted in a study by Trovato Salinaro et al. (2014). In fact, both AD and glaucoma have a common pathogenetic role played by free radicals and the ability to activate the adaptive stress response systemically (Trovato Salinaro et al., 2014). As in many cells, the activation of cell stress response is a response mechanism to the damage caused by free radicals and its modulation by nutritional supplements, defined nutraceuticals, is currently considered an adjuvant approach to standard therapy of diseases, including AD (Mecocci et al., 2014). The results by Scuderi et al. (2014) seem to support this theory, showing that the up-regulation of the sirtuin system, *via* resveratrol, is able to reduce the activation of astrocytes and the production of inflammatory mediators in primary cultures of rat astrocytes treated with Aß (1–42). The causal role of infectious agents in relation to the pathogenesis of AD has been little experimented. As reported by Piacentini et al. (2014), recurrent infection by herpes simplex virus type I (HSV-1) can be considered a risk factor for AD. The mechanism(s) through which HSV-1 could give rise to AD are not completely understood, even if the HSV-1–mediated proteolytic processing of APP, generating also Aβ 40 and Aβ 42 monomers or small oligomers, is among those which received the greatest consensus (Piacentini et al., 2014).

Among the issues with the highest translational impact on research in the field of AD is the use of “old” drugs such as vitamin D, currently used for years in the treatment of bone loss, above all in the elderly, whose neuroprotective properties in subjects with AD have been recently brought to light (Annweiler et al., 2014). A very updated and comprehensive review article by Cornelius et al. (2014) examines the factors that can be considered as a common denominator of AD and osteoporosis. The dysregulation of the endocannabinoid system, initially based on a number of preclinical evidence, as a viable mechanism to intervene in the pathogenesis of AD is no longer controversial (Aso and Ferrer, 2014). Furthermore, the availability of “new” pharmacological tools to modulate the endocannabinoid system of the brain, one with the preclinical evidence of their effectiveness in terms of a significant reduction of oxidative and metabolic damage load on neurons and objective improvements of behavior in individuals with AD, leads research further toward the study of the endocannabinoid system as a novel therapeutic target in AD (Aso and Ferrer, 2014). An example of how a virtuous collaboration between non-profit institutions, such as universities, and pharmaceutical companies should develop is in the article by Lundkvist et al. (2014). This paper focuses on the activities of “AlzeCure,” a non-profit Swedish foundation founded by brilliant researchers with a strong background in R&D from a Big Pharma such as Astra Zeneca. The AlzeCure acts as a catalyst, receiving continuous feedback from biotech companies, and leading academic institutions (e.g., Karolinska Institute) involved in R&D of drugs for AD, collaborating in the preclinical and clinical field, demonstrating that a useful collaboration between non-profit institutions and pharmaceutical companies is actually possible (Lundkvist et al., 2014). A viable alternative to this model of academic-industry cooperation is the one proposed by Beauchet et al. (2014) who, in their Commentary, described the activities of “Biomathics,” an emerging scientific research consortium whose task is to promote cooperation among the academic research teams working in the field of aging and longevity. One of the main goals of this Consortium is to foster the creation of large research groups, formed by scientists worldwide, so as to increase the possibility of applying and obtaining research grants to enhance international basic research (Beauchet et al., 2014).

## Conflict of interest statement

The authors declare that the research was conducted in the absence of any commercial or financial relationships that could be construed as a potential conflict of interest.
